# Heterologous Expression of a Thermostable β-1,3-Galactosidase and Its Potential in Synthesis of Galactooligosaccharides

**DOI:** 10.3390/md16110415

**Published:** 2018-10-30

**Authors:** Haitao Ding, Lili Zhou, Qian Zeng, Yong Yu, Bo Chen

**Affiliations:** SOA Key Laboratory for Polar Science, Polar Research Institute of China, Shanghai 200136, China; htding@outlook.com (H.D.); lilizhou1199@163.com (L.Z.); zengqianmu@126.com (Q.Z.); yuyong@pric.org.cn (Y.Y.)

**Keywords:** β-galactosidase, recombinant, thermostable, transglycosylation, galactooligosaccharides, *Marinomonas*

## Abstract

A thermostable β-1,3-galactosidase from *Marinomonas* sp. BSi20414 was successfully heterologously expressed in *Escherichia coli* BL21 (DE3), with optimum over-expression conditions as follows: the recombinant cells were induced by adding 0.1 mM of IPTG to the medium when the OD_600_ of the culture reached between 0.6 and 0.9, followed by 22 h incubation at 20 °C. The recombinant enzyme β-1,3-galactosidase (rMaBGA) was further purified to electrophoretic purity by immobilized metal affinity chromatography and size exclusion chromatography. The specific activity of the purified enzyme was 126.4 U mg^−1^ at 37 °C using ONPG (*o*-nitrophenyl-β-galactoside) as a substrate. The optimum temperature and pH of rMaBGA were determined as 60 °C and 6.0, respectively, resembling with its wild-type counterpart, wild type (wt)MaBGA. However, rMaBGA and wtMaBGA displayed different thermal stability and steady-state kinetics, although they share identical primary structures. It is postulated that the stability of the enzyme was altered by heterologous expression with the absence of post-translational modifications such as glycosylation, as well as the steady-state kinetics. To evaluate the potential of the enzyme in synthesis of galactooligosaccharides (GOS), the purified recombinant enzyme was employed to catalyze the transgalactosylation reaction at the lab scale. One of the transgalactosylation products was resolved as 3′-galactosyl-lactose, which had been proven to be a better bifidogenic effector than GOS with β-1,4 linkage and β-1,6 linkages. The results indicated that the recombinant enzyme would be a promising alternative for biosynthesis of GOS mainly with β-1,3 linkage.

## 1. Introduction

Galactooligosaccharides (GOS) are non-digestible oligosaccharides composed of 3–10 galactosyl groups and a terminal glucose [1]. As an important type of dietary supplement, GOS is difficult to digest by the gastrointestine directly, whereas it can specifically stimulate the growth of the prebiotics inhabited in the intestine, such as *Lactobacillus* and *Bifidobacteria*, rather than maleficent bacteria [2]. It is well known that the growth of probiotics can improve immunity and prevent cancer [3]. As a matter of fact, it is impossible to obtain enough natural GOS from milk to satisfy the increasing demanding due to the low amount of GOS in milk [4].

To provide sufficient GOS for humans, chemical and enzymatic approaches have been developed to synthesis of GOS in practice [5]. However, because of a lack of specificity of the product and the extreme condition for hydrolysis of lactose to generate monosaccharides, chemical methods are not utilized on a large scale. In contrast, enzymatic synthesis of GOS exhibits good stereoselectivity and regioselectivity, with mild reaction conditions [6]. Thus, GOS present in the market are all produced from lactose by employing various β-D-galactosidases (E.C. 3.2.1.23, BGAs) [7].

BGAs could be produced by a great number of organisms, including microorganisms, and animal and plant cells [8]. All BGAs are capable of catalyzing the hydrolysis of the β-glycosylic linkage of lactose to generate glucose and galactose, as well as transferring of the galactose onto the galactose moiety of lactose to yield GOS [6]. Generally, the hydrolytic activity of BGA has been employed to remove lactose from milk for people with lactose intolerance, while the transgalactosylation activity has been developed for the production of diverse functional galactosylated products, especially prebiotics like GOS [9].

BGAs catalyze two forms of transglycosylation reactions, including intramolecular and intermolecular reactions. The former involves the direct transfer of galactosyl groups to glucose to produce lactose isomers, such as allolactose, which formed β-1,6 linkage near the end of the hydrolysis reactions, before the glycosyl molecules diffuse out of the active site. Unlike the intramolecular reaction, the intermolecular reaction can produce disaccharides, trisaccharides, tetrasaccharides, and polysaccharides with a higher degree of polymerization. During the catalytic reaction of β-galactosidase, the synthesized GOS can also serve as a substrate for the hydrolysis reaction, resulting in dynamic changes in the components and amounts of GOS [10].

As a rule of thumb, thermal stability is imperative for the application of enzymes in practice. To obtain quantified BGAs for GOS manufacturing, BGAs from various sources had been extensively studied [11,12,13]. In our previous study, a novel β-1,3-galactosidase (MaBGA) from *Marinomonas* sp. BSi20414, a strain isolated from the Arctic Ocean, has been purified and characterized [14]. This enzyme showed robust thermal stability and strict substrate specificity toward β-1,3 linkage, providing a competitive candidate for biosynthesis of GOS with β-1,3 linkage. In the present work, the enzyme MaBGA was heterologously expressed in *Escherichia coli* and the purified enzyme was subsequently employed to catalyze the transglycosylated reaction to generate oligosaccharides, for evaluating the potential application of MaBGA in biosynthesis of GOS.

## 2. Results

### 2.1. Construction of Recombinant Cells

The gene *mabga* was amplified from *Marinomonas* sp. BSi20414 using the corresponding primer pairs. The purified PCR product was digested with restriction endonucleases *Nde*I and *Xho*I, as well as plasmid pET-22b (+). The digested PCR product and vector were ligated together to construct recombinant plasmid. The recombinant plasmid was transformed into *E. coli* DH5α and the positive clones were picked for sequencing. Subsequently, the verified plasmid with correct insert sequence was transformed into *E. coli* BL21 (DE3) for expression.

### 2.2. Optimization of the Expression Condition of rMaBGA

**Figure 1:**
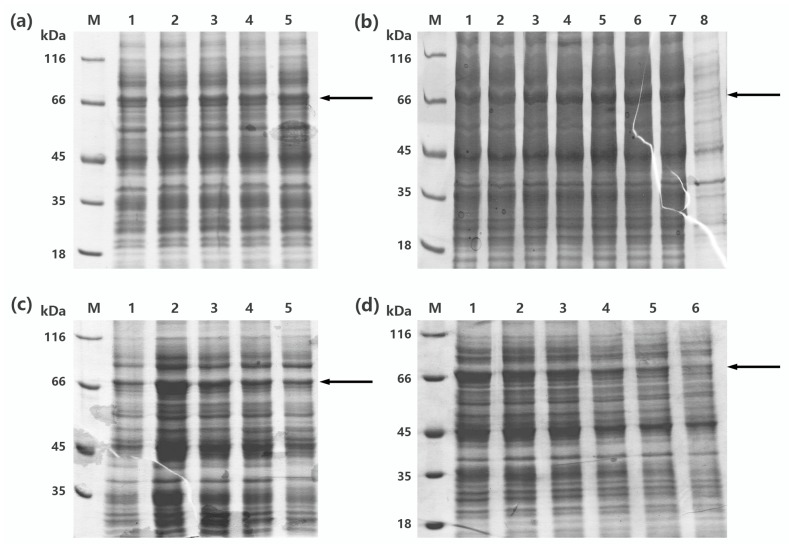
SDS-PAGE (sodium dodecyl sulfate polyacrylamide gel electrophoresis) analysis of the production of the recombinant enzyme β-1,3-galactosidase (rMaBGA) under different expression conditions. (**a**) The supernatant of cell lysates induced at different OD. Lane 1–5: OD reached 0.2, 0.6, 0.7, 0.9, and 1.3, respectively. (**b**) The supernatant of cell lysates induced with different IPTG concentration. Lane 1–8: the IPTG concentration was 0.05, 0.1, 0.3, 0.5, 0.7, 0.9, 1.2, and 0 mM, respectively. (**c**) The supernatant of cell lysates induced at different temperatures. Lane 1–5: the induced temperature was set to 15, 20, 25, 30, and 37 °C, respectively. (**d**) The supernatant of cell lysates induced for different time. Lane 1–6: the induced time was 22, 10, 8, 7, 4, and 2 h, respectively. Lane M: protein molecular weight marker. The location of rMaBGA was marked with black arrows.

To obtain more soluble recombinant protein, one-factor-at-a-time design was implemented for optimization of the expression condition of recombinant MaBGA (rMaBGA), using cell density, concentration of inducer, temperature, and duration for induction as four variables. According to the soluble expression of rMaBGA under different expression conditions (Figure 1), examined by SDS-PAGE (sodium dodecyl sulfate polyacrylamide gel electrophoresis), the optimized expression condition was adopted as follows: the recombinant cells were induced by adding 0.1 mM of IPTG to the medium when the OD_600_ of the culture reached between 0.6 and 0.9, followed by 22 h incubation at 20 °C.

### 2.3. Purification of rMaBGA

**Figure 2:**
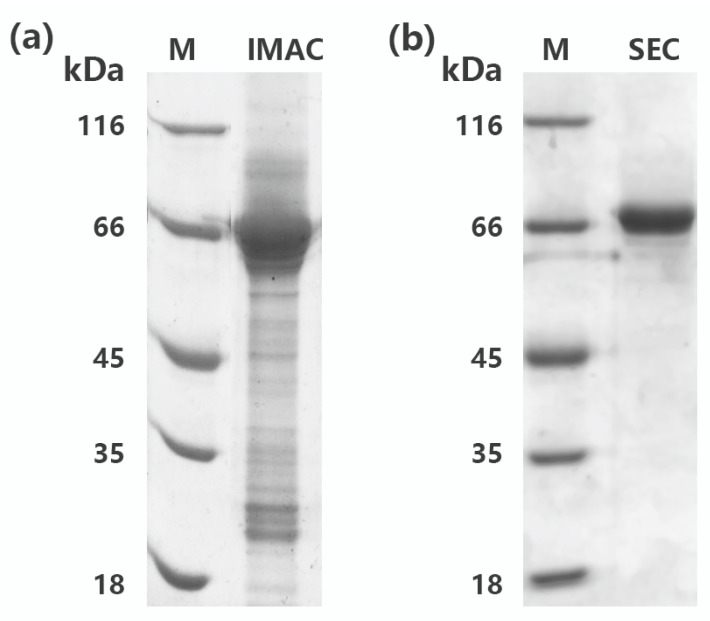
SDS-PAGE analysis of rMaBGA first purified by immobilized metal affinity chromatography (IMAC) (**a**) and then purified by size exclusion chromatography (SEC) (**b**). Lane M: protein molecular weight marker. Lane IMAC: rMaBGA purified by IMAC. Lane SEC: rMaBGA purified by IMAC and SEC, sequentially.

In general, most recombinant protein containing 6X His-tag could be easily purified to electrophoretic purity by one-step immobilized metal affinity chromatography (IMAC). However, rMaBGA cannot be purified to a single band after loading onto a column packed with Ni-NTA agarose, as shown on the gel in Figure 2a. Therefore, the elute of IMAC was further purified by size exclusion chromatography (SEC). As expected, the purified enzyme was represented as a homogeneous band corresponding to 66 kDa on the gel (Figure 2b), indicating that rMaBGA had been purified to electrophoretic purity. The specific activity of the purified rMaBGA was 126.4 U mg^−1^ at 37 °C using ONPG (*o*-nitrophenyl-β-galactoside) as substrate.

### 2.4. Enzymatic Characterization of MaBGA

#### 2.4.1. Effect of pH and Temperature on the Activity of rMaBGA

**Figure 3:**
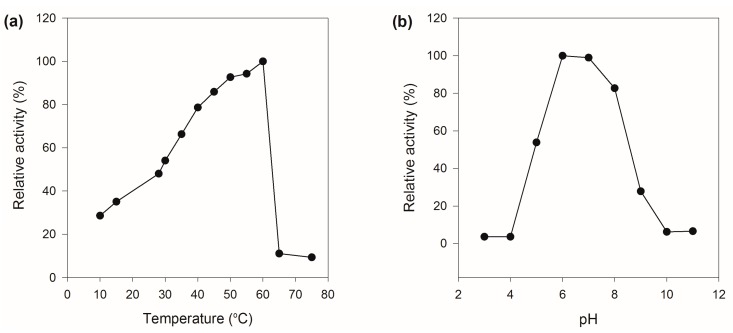
Effects of temperature and pH on the activity of rMaBGA. (**a**) Effect of temperature on the activity of rMaBGA; (**b**) effect of pH on the activity of rMaBGA.

The recombinant MaBGA showed its maximum activity at 60 °C (Figure 3a), as well as the wild type MaBGA (wtMaBGA). Although rMaBGA and wtMaBGA displayed a similar temperature-activity relationship at temperatures below 60 °C, the activity of rMaBGA dropped to 10% of its highest activity at 65 °C, whereas wtMaBGA still retained nearly 80% of its highest activity at the same temperature. The optimum pH of rMaBGA was determined as 6.0 (Figure 3b), resembling wtMaBGA. Moreover, the pH-activity profile of rMaBGA was also similar to that of wtMaBGA.

#### 2.4.2. Thermal Denaturation Kinetic of rMaBGA

As shown in Table 1, the half-life at 50 °C and 60 °C of wtMaBGA was 1.66 and 2.42 times more, respectively, than those of rMaBGA, indicating that rMaBGA was less stable than wtMaBGA, also indicated by the values of inactivation enthalpy (Δ*H*), inactivation free energy (Δ*G*), and inactivation entropy (Δ*S*).

#### 2.4.3. Steady-State Kinetic of rMaBGA

The Michaelis–Menten constant *K_m_* and the maximum reaction velocity *V_max_* of rMaBGA were determined as 6.85 mM and 64.13 μM min^−1^ (Table 2), respectively, using a nonlinear fitting plot. However, the values of *K_m_* and *V_max_* for the recombinant MaBGA showed a significant difference from those of its wild type counterpart, which were measured as 14.19 mM and 1.05 μM min^−1^, respectively, in our previous study [14].

### 2.5. Synthesis of Galactooligosaccharides

#### 2.5.1. Thin-Layer Chromatography Analysis

**Figure 4:**
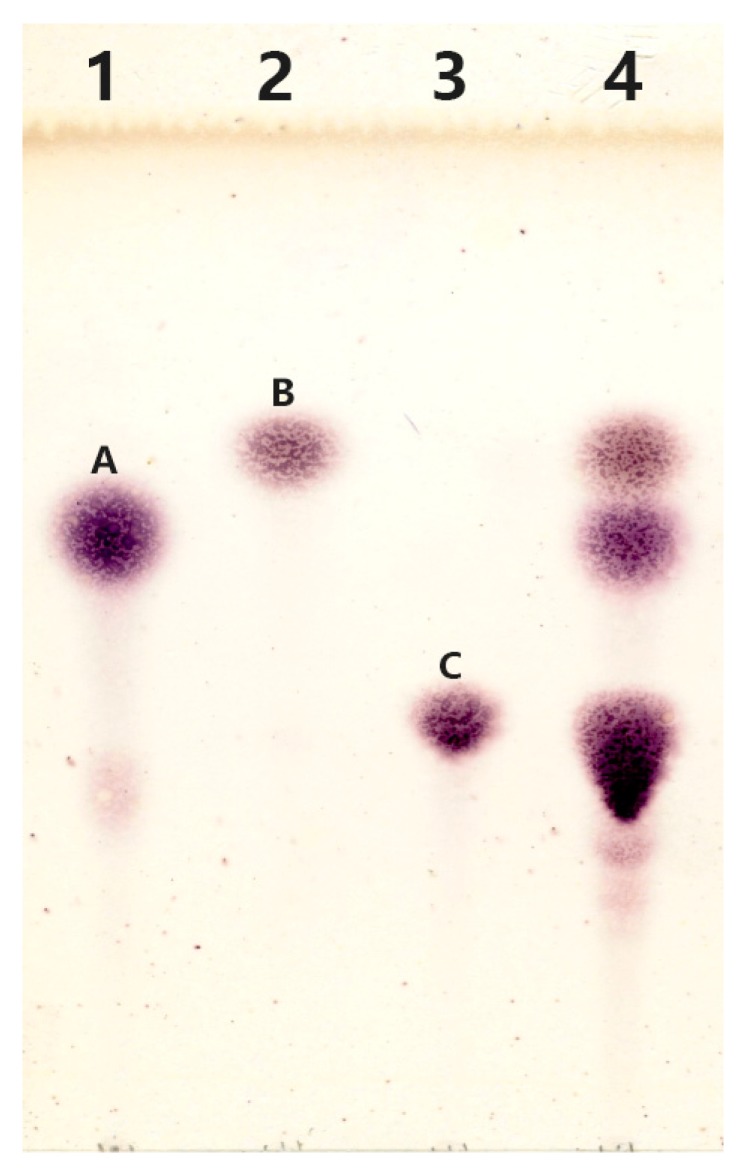
Analysis of transgalactosylation activity of rMaBGA by TLC (thin layer chromatography). Lane 1: galactose (spot A); Lane 2: glucose (spot B); Lane 3: lactose (spot C); Lane 4: the products of lactose catalyzed by rMaBGA.

The reaction mixture was subjected to thin-layer chromatography analysis after removal of the enzyme. It is obvious that the reaction mixture (Figure 4) contained spots corresponding to spots A (galactose), B (glucose), and C (lactose), indicating the occurrence of the hydrolysis reaction. In addition, several blurry spots can be found from Lane 4 at the position below spot C. It is supposed that the substance represented as blurry spots might be the products of transglycosylation reaction catalyzed by rMaBGA.

#### 2.5.2. Characterization of the Products of Transglycosylation

The transglycosylation products were purified and separated into three components, designated as LLZ-01, LLZ-02, and LLZ-03, using gel chromatography and HPLC. Based on the ESI-MS and NMR analyses, component LLZ-01 was characterized as a mixture of α-lactose and β-lactose with ratios between 1:7 and 1:8, whereas component LLZ-02 was determined to be the mixture of α-lactose/β-lactose and trisaccharides with a ratio of 1:1. Unfortunately, the low purity of LLZ-03 resulted in complex MS and NMR signals, which was difficult to resolve. Additional purification steps are indispensable for characterization of LLZ-03 in further studies.

**Figure 5:**
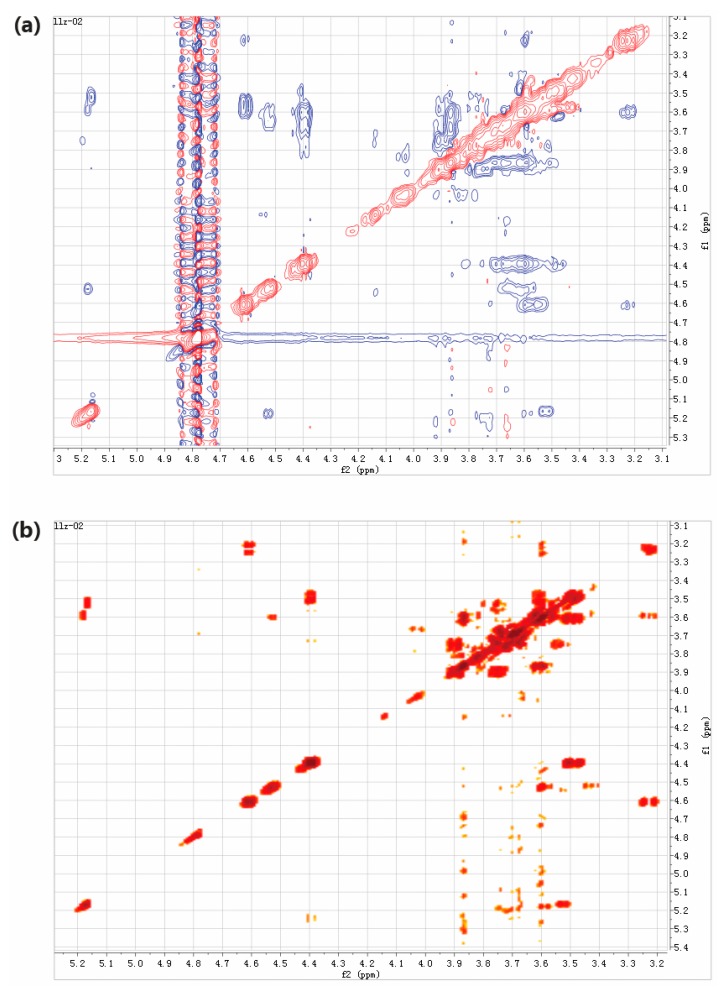
Two-dimensional NMR analysis of LLZ-02. (**a**) ^1^H-^1^H COSY spectrum. (**b**) NOESY spectrum.

**Figure 6:**
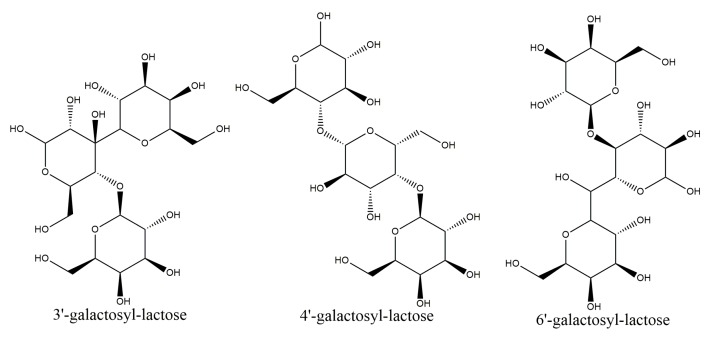
Molecular structures of 3′-galactosyl-lactose, 4′-galactosyl-lactose, and 6′-galactosyl-lactose.

In the present study, two-dimensional NMR techniques, ^1^H-^1^H COSY and NOESY were adopted to resolve structure of the trisaccharides presented in LLZ-02 (Figure 5), using trisaccharide *O*-β-D-galactopyranosyl-(1-4)-*O*-β-D-galactopyranosyl-(1-4)-D-glucopyranose (4′-galactosyl-lactose) as reference in the analysis. All the chemical shifts were tabulated in Table 3 according to the signal of ^1^H-^1^H COSY. Clear NOE was detected between H1 of β-galactosyl-A (4.37 ppm) and H4 of α-glucosyl (3.50 ppm)/β-glucosyl (3.46 ppm), as well as between H1 of β-galactosyl-B (4.50 ppm) and H3 of β-glucosyl (3.42 ppm). It is suggested that the linking between β-galactosyl-A and α-glucosyl/β-glucosyl occurred at the 1,4 sites, and the linking between β-galactosyl-B and β-glucosyl occurred at the 1,3 sites. Therefore, the trisaccharide of LLZ-02 was determined as β-D-galactosyl-(1-3)-β-D-galactosyl-(1-4)-D-glucose (3′-galactosyl-lactose, Figure 6).

## 3. Discussion

In this study, a thermostable β-1,3-galactosidase from *Marinomonas* sp. BSi20414 was successfully heterologously expressed in *Escherichia coli* BL21 (DE3), with optimized over-expression conditions. The purified recombinant enzyme was characterized biochemically and employed to the synthesis of GOS, which were further analyzed by ESI-MS and NMR to resolve the molecular structures.

Although rMaBGA displayed similar optimum catalytic pH and temperature to those of its wild-type counterpart, these two-form enzymes, which share an identical primary structure, exhibited different thermal stability and steady-state kinetics. Beyond expectations, the half-life at 50 °C and 60 °C of wtMaBGA were 1.66 and 2.42 times more, respectively, than those of rMaBGA. The decrease in values of ΔH and ΔG of both wtMaBGA and rMaBGA was concomitant with increase in the temperature, suggesting that the conformation of the protein was altered changed by heat treatment. The higher values of Δ*G* for wtMaBGA than those for rMaBGA indicated that wtMaBGA showed more resistance against thermal denaturation than rMaBGA at the same temperature, also suggested by the increase of entropy of inactivation (Δ*S*), which is often accompanied by disruption of enzyme structure. As rMaBGA and wtMaBGA share exactly the same amino acid sequences, it is supposed that the difference in stability and kinetics between them might be caused by the post-translational modification (PTM) occurring in the wild-type strain. PTM, such as glycosylation [15], phosphorylation [16], and methylation [17], affects the kinetic, stability, and structural features of proteins through regulating their biophysical characteristics. Several studies reported that the glycosylated proteins exhibited higher stability, which have higher melting temperature and greater free energy than their non-glycosylated wild-type counterpart [18]. Therefore, it is postulated that the stability of the enzyme was altered by heterologous expression with the lack of PTM, as well as the steady-state kinetics. Although the recombinant MaBGA was less stable than its wild-type counterpart, its thermal stability is still qualified for practical application. Every coin has two sides, the recombinant enzyme showed superior catalytic activity than the wild-type form, as the *V_max_* of the former was 61-fold higher than that of the latter.

Generally, β-galactosidases from various sources produce diverse GOS mixtures with different degrees of polymerization (DP) and glycosidic linkages. For instance, β-galactosidase from *Bacillus circulans* produces predominantly β-1,4 linked GOS, while β-galactosidase from *Kluyveromyces lactis* mainly forms GOS with β-1,6 linkage [4,19], whereas the dominant transglycosylation product in this study was identified as trisaccharide with β-1,3 linkages. Regardless of the fact that β-1,4 and β-1,6 are common linkages in GOS, β-1,3 linkage is relatively rare [20]; the latter showed stronger prebiotic effect than the former. Previous studies have shown that GOS containing mainly β-1,3 linkage had a better bifidogenic effect than GOS containing mainly β-1,4 and β-1,6 linkages after one week of intake by healthy humans [21]. Therefore, owing to its soluble over-expression, thermal stability, and selectivity toward β-1,3 linkage, the recombinant MaBGA was proven to be a promising alternative for biosynthesis of 3′-galactosyl-lactose, a probiotic with better bifidogenic effect.

## 4. Materials and Methods

### 4.1. Expression and Purification of rMaBGA

#### 4.1.1. Strains, Plasmids, and Culture Conditions

Strain *Marinomonas* sp. BSi20414, isolated from the Arctic Ocean [22], was used as the source of β-galactosidase. *Escherichia coli* DH5α and BL21 (DE3), cultivated in Luria–Bertani medium at 37 °C, were used for gene cloning and expression, respectively. Plasmid pET22b (+) was employed to construct recombinant plasmid. All chemicals were of analytical grade.

#### 4.1.2. Construction of Recombinant Strains

The gene encoding for MaBGA was amplified using DNA of *Marinomonas* sp. BSi20414 as a template with the forward primer 5′-GGAATTCCATATGAAGTTAGGTGTATGTTACTACC-3′ and the reverse primer 5′-GTTCGCGCTCGAGGATTTCTTGCCAAATGGC-3′, carrying the cleavage sites of *Nde*I and *Xho*I (underlined), respectively. The PCR product was digested with restriction endonucleases *Nde*I and *Xho*I simultaneously, and ligated with the vector pET-22b (+) digested by the same enzymes. The constructed plasmid was transformed into *E. coli* DH5α competent cells for sequencing. Subsequently, the verified plasmid harboring the desired gene was transformed into *E. coli* BL21 (DE3) competent cells for expression.

#### 4.1.3. Optimization of the Production of rMaBGA

Cells were cultivated in Luria–Bertani broth at 37 °C with 100 μg mL^−1^ ampicillin added. Subsequently, single-factor experimental design was employed for the optimization of the production of rMaBGA. Soluble expression of rMaBGA was estimated under different cell density, IPTG concentration, incubation temperature, and time adopted for induction.

#### 4.1.4. Expression and Purification of rMaBGA

The expression of the rMaBGA was performed according to the optimization procedure, as follows: the induction of the recombinant cells was started by adding 0.1 mM IPTG to the broth when the OD_600_ of the culture reached between 0.6 and 0.9, followed by 22 h incubation at 20 °C. Cultures were collected by centrifugation at 10,000× *g* for 10 min. The precipitate was washed and suspended with lysis buffer (50 mM sodium phosphate buffer, 10 mM imidazole, 200 mM NaCl, 0.5% glycerol, pH 7.0). Cells were then lysed by ultrasonication (burst of 5 s followed by intervals of 10 s repeated 90 times). The cell debris was discarded by centrifugation at 10,000× *g* for 15 min and the crude enzyme was loaded onto a column packed with Ni-NTA resin. The resin was washed with wash buffer (50 mM sodium phosphate buffer, 20 mM imidazole, 200 mM NaCl, 0.5% glycerol, pH 7.0) and subsequently eluted with elution buffer (50 mM sodium phosphate buffer, 250 mM imidazole, 200 mM NaCl, 0.5% glycerol, pH 7.0). The eluted enzyme was concentrated by ultrafiltration and then subjected to a prepacked gel filtration column filled with Surperdex G200 with a flow rate of 0.4 mL min^−1^. The eluted enzyme was desalted and concentrated by ultrafiltration and stored at −80 °C. All purification steps were implemented at 4 °C. The protein concentration was measured by Bradford method using bovine serum albumin (BSA) as a standard [23].

#### 4.1.5. SDS-PAGE Analysis

The purified rMaBGA was examined by SDS-PAGE running on a 5% stacking gel and an 8% separating gel [24]. Gel was stained with Coomassie Brilliant Blue R-250. The molecular weight of rMaBGA was determined using protein molecular weight marker (MBI) as a reference.

### 4.2. Enzymatic Characterization of rMaBGA

#### 4.2.1. Enzyme Activity Assay

The enzyme activity was measured by monitoring the absorbance of ONP (*o*-nitrophenyl) at 420 nm in 50 mM PBS buffer (pH 7.0) at 37 °C, with 10 mM of ONPG was used as substrate. The concentration of ONP was obtained from the standard curve. One unit of β-galactosidase activity was defined as the amount of enzyme demanded for catalyzing the formation of 1 μmol ONP per minute.

#### 4.2.2. Effect of pH and Temperature on the Activity of rMaBGA

The optimum pH for rMaBGA was measured by assaying its activity with different pH ranging from 3.0 to 11.0 in Britton–Robinson buffer. The optimum temperature for rMaBGA was measured by assaying its activity at different temperatures from 10 to 70 °C.

#### 4.2.3. Thermal Denaturation Kinetic of rMaBGA

The thermal stability of rMaBGA was evaluated by measuring the remaining activity after incubation of the enzyme at 50 °C and 60 °C for 1 h with 5 min intervals. Thermodynamic parameters for thermal unfolding of rMaBGA were calculated according to Ding et al. [25].

#### 4.2.4. Steady-State Kinetic of rMaBGA

The activity of rMaBGA was assayed with different concentrations of ONPG ranging from 0.25 to 5 mM to analyze the steady-state kinetic of the enzyme. The kinetic parameters were calculated by nonlinear fitting of the Michaelis–Menten Equation (1):

*v* = *V_max_* [S]/(*K_m_* + [S]) (1)

where *K_m_* and [S] are Michaelis constants and concentration of ONPG, respectively.

### 4.3. Synthesis of Galactooligosaccharides

#### 4.3.1. Thin-Layer Chromatography Analysis

The transglycosylation reaction was conducted at 40 °C for 5 h by adding 3.5 U rMaBGA to 50 mM PBS buffer (pH 7.0) containing 480 mM lactose. Subsequently, the enzyme was inactivated by boiling for 2 min. The denatured enzyme was removed by centrifugation at 10,000× g for 15 min and the supernatant was used for thin-layer chromatography analysis. A solvent mixture consisting of 50% n-butanol, 20% ethanol, and 30% water was employed to develop the TLC plate in a developing chamber. The visualization reagents contained 0.5% 3,5-dihydroxytoluene and 20% H_2_SO_4_.

#### 4.3.2. Characterization of the Products of Transglycosylation

The supernatant prepared as described above was concentrated by rotary evaporation and dissolved by 50% ethanol, which was then subjected to a prepacked column filled with Sephadex LH-20 and eluted with 50% ethanol. The elute was collected by a fraction collector and was analyzed by TLC. Fractions containing transglycosylation product were combined and concentrated by rotary evaporation. The concentrate was dissolved by the appropriate amount of water and filtered through a 0.22 µm membrane. Subsequently, the concentrated products were separated by HPLC (LC-10AdvP, SHIMADZU, Kyoto, Japan) equipped with a semi-preparation YMC-Pack NH2 column, with a flow rate of 3 mL min^−1^ and temperature of 30 °C. The elution peaks were concentrated by rotary evaporation, then dissolved by appropriate amount of water, and further characterized by MS and NMR.

## 5. Conclusions

In the present work, a thermostable β-1,3-galactosidase from *Marinomonas* sp. BSi20414 was successfully heterologously expressed in *Escherichia coli* BL21 (DE3), with optimized over-expression conditions. The recombinant enzyme was further purified to electrophoretic purity and characterized biochemically. Although rMaBGA showed similar profiles of optimum catalytic pH and temperature to those of its wild-type counterpart, these two-form enzymes, which share an identical primary structure, exhibited different thermal stability and steady-state kinetics. It is assumed that the stability and the steady-state kinetics of the enzyme were altered by heterologous expression with the absence of post-translational modifications such as glycosylation. Furthermore, owing to the soluble over-expression, thermal stability, and selectivity toward β-1,3 linkage, rMaBGA was proven to be a promising alternative for biosynthesis of 3′-galactosyl-lactose, a probiotic with better bifidogenic effect than GOS with β-1,4 linkage and β-1,6 linkages.

## Figures and Tables

**Table 1:** Thermodynamics of irreversible thermal denaturation of β-1,3-galactosidase (MaBGA). wt—wild type; r—recombinant enzyme.

Enzyme	Temperature (°C)	*k*_d_ (h^−1^)	t_1/2_ (h)	Δ*H* (KJ mol^−1^)	Δ*G* (KJ mol^−1^)	Δ*S* (J mol^−1^ K^−1^)
wtMaBGA	50	0.0433	16.00	114.03	87.75	81.35
	60	0.1597	4.34	113.94	86.94	81.10
rMaBGA	50	0.0721	9.61	147.75	86.38	189.99
	60	0.3879	1.79	147.66	84.48	189.74

**Table 2:** Kinetic constants of rMaBGA.

Enzyme	*K_m_* (mM)	*V_max_* (μM min^−1^)
rMaBGA	6.85	64.13
wtMaBGA	14.19	1.05

**Table 3:** Proton chemical shifts for LLZ-02.

Unit	α-glucosyl	β-glucosyl	β-galactosyl-A	β-galactosyl-B
1	5.14	4.59	4.37	4.50
2	3.52	3.21	3.62	3.62
3	3.79	3.42	3.84	3.70
4	3.50	3.46	4.01	3.86
5	3.85	3.60	3.70	3.67
6	3.75–3.88	3.75–3.88	3.70–3.85	3.70–3.85
